# Addictive behavior and suicidality in patients followed by the department of psychiatry in the region of southern tunisia

**DOI:** 10.1192/j.eurpsy.2021.1564

**Published:** 2021-08-13

**Authors:** A. Kerkeni, W. Abbes, A. Frikha, K. Medhaffer, M. Abbes, K. Zitoun, L. Ghanmi

**Affiliations:** The Department Of Psychiatry, Hospital of gabes, Gabes, Tunisia

**Keywords:** Addictive behavior, Common vulnerability factors, Suicidality

## Abstract

**Introduction:**

Addictive behaviors and suicide have important risks that need to be explored for any patient followed at the department of psychiatry, possibly endangering his vital and psychosocial prognosis.

**Objectives:**

Our study aims to identify the link between addictive behavior and suicidal behavior in patients followed at the psychiatry department at the regional hospital of Gabes.

**Methods:**

We conducted a cross-sectional, descriptive and analytical study carried out on a clinical population who consult in the psychiatry department in the Gabes’s regional hospital during the period from January 1st, 2020 to September 30, 2020.Sociodemographic and clinical data of the patients as well as their personal and family history were assessed. The evaluation of psychopathological disorders was carried out according to the diagnostic criteria of DSM-5. Suicide Behaviors Questionnaire (SBQ) was used for suicide risk assessment. Fagerstrom questionnaire in its validated French version was used to assess the of nicotine dependence. The exploration of childhood physical and emotional trauma was carried out by the Childhood Trauma Questionnaire (CTQ) scale. Data were analysed using the software SPSS.

**Results:**

100patients were included. The mean age was 45.5years. TableI: Breakdown of addictive behavior and suicide attempts by gender. TableII: Common vulnerability factors between addictive behavior and suicidality

**Figure:**
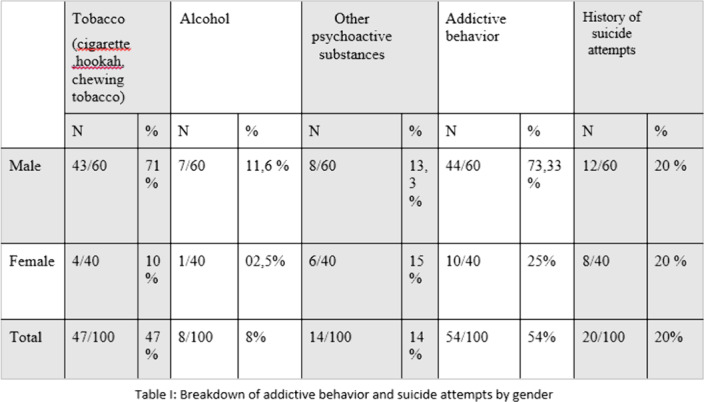

**Figure:**
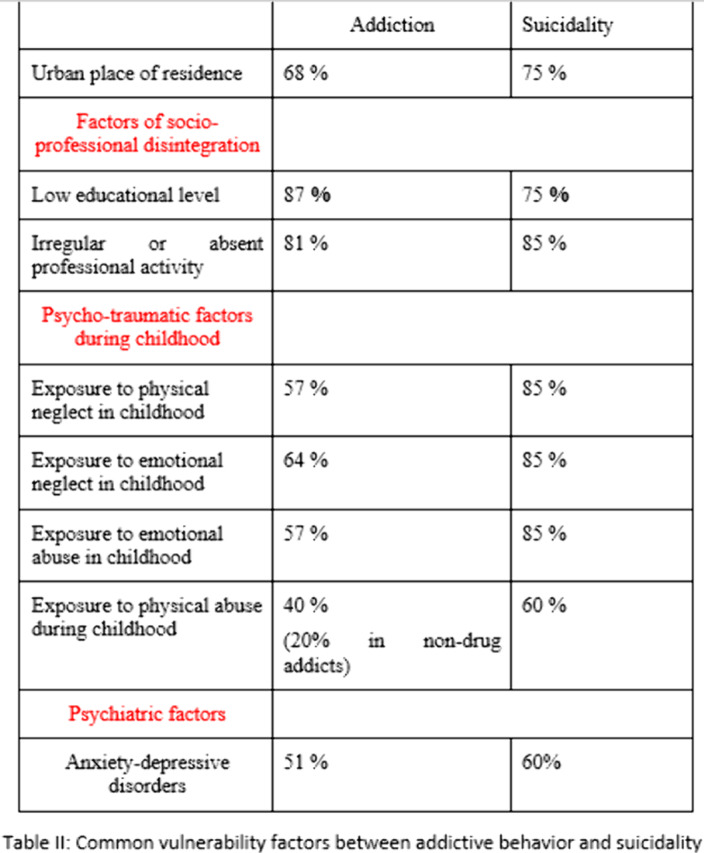

The analytical study showed that suicide attempts were correlated with addictive behaviors (p = 0.03) and that suicidal recurrence was correlated with addictive behaviors (p = 0.01).

**Conclusions:**

Suicidal behavior in patients followed in psychiatry is closely linked to addictive behavior, hence the importance of early management.

